# The Role of Structured Exercise in Breast Cancer Survivorship: A Prospective Observational Study

**DOI:** 10.7759/cureus.87272

**Published:** 2025-07-04

**Authors:** Baraah M Mohamed, Christina M Paluskievicz, Caitlin Beane, Nancy Buderer, Lisa Jacobs, Olutayo A Sogunro

**Affiliations:** 1 General Surgery, York Wellspan Hospital, York, USA; 2 Department of Surgery and Division of Breast Surgical Oncology, University of Maryland, College Park, USA; 3 Department of Surgery and Division of Breast Surgical Oncology, Johns Hopkins Hospitals, Baltimore, USA; 4 Department of Statistics, Nancy Buderer Consulting, LLC, Toledo, USA

**Keywords:** breast cancer survivorship, cancer rehabilitation, exercise adherence, exercise intervention, physician-endorsed exercise, post-treatment exercise, quality of life, structured exercise program

## Abstract

Introduction

Cancer survivorship is defined as the time from the diagnosis of cancer and throughout the remainder of a patient's life. Survivorship care is a comprehensive approach to optimizing health maintenance after completing primary cancer treatment. Transitioning to survivorship care in breast cancer patients includes focusing on the long-term effects of cancer, its treatment, diet, and lifestyle modification. It is known that exercise during and after cancer treatment plays an essential role in preventing multiple cancer health-related outcomes such as fatigue, anxiety, and depression. However, most cancer patients remain inactive during and beyond cancer treatment due to the lack of clear recommendations for a physician-endorsed exercise program. Our pilot interventional study investigates the effect of a structured exercise program on breast cancer survivorship and quality of life when exercise is endorsed by the treating physician.

Design

This is a prospective observational study that included female breast cancer patients aged 18 and over who completed their active breast cancer treatment six to 12 months prior. Those who consented were enrolled in a structured exercise program when it was explicitly endorsed by their primary oncologic physician. The structured program was offered by a third-party not-for-profit organization. It included evidence-based exercises recommended for cancer patients, led by a certified cancer fitness instructor for 10 weeks, targeting strength, resistance training, cardio, balance, and flexibility. The patients consented to complete a quality-of-life survey (SF-36) before and after the program. The primary outcome of interest included improvement in overall quality of life. The secondary outcomes of interest included compliance rates to completion of the exercise program and long-term symptom improvements.

Results

A total of five patients consented to the study. Overall, there was a significant improvement in various components of quality of life. These improvements were observed in their physical functioning (mean change 13.0, 95% confidence interval (CI): 5.9 to 20.1, P < 0.05), energy/fatigue (mean change 16.0, CI: 2.5 to 29.5, P < 0.05), emotional well-being (mean change 9.6, CI: 1.3 to 17.9, P < 0.05), and social functioning (mean change 12.5, 1.5 to 23.5, P < 0.05). The five female breast cancer survivors completed the exercise program with a compliance rate of 100%. There seemed to be an increase in a sense of community and accountability developed between the participants during the exercise period.

Conclusion

Participating in a structured exercise program when exercise was specifically endorsed by an oncologic physician significantly improves the quality of life of breast cancer patients. Participants had improved physical functioning, energy, fatigue, emotional well-being, and social functioning, which are essential components of well-optimized survivorship care. Also, when regular exercise is endorsed explicitly by a treating physician, there are high compliance rates. Moreover, we demonstrated the feasibility of investigating the impact of a structured exercise program on breast cancer patients, with the aim of expanding to a larger population size with a control group in the future for additional insights.

## Introduction

Over the last several decades, the number of breast cancer survivors has increased in the United States due to early detection, better treatment options, and improved overall awareness [1]. With the improving survival rate, there is a growing need for optimized care, including endorsed programs to support the survivors [2]. Cancer survivorship is the state of living requiring comprehensive care from the time of cancer diagnosis to optimize health after completing the primary cancer treatment. This comprehensive care is defined as survivorship care, which focuses on the long-term effects of cancer, including physical and psychosocial changes, diet, and lifestyle modification. In the follow-up setting, breast cancer survivorship is one of the most challenging topics to address, as breast cancer is often linked to many relevant and complex physical and psychosocial alterations affecting quality of life [3-7].

Studies have shown that breast cancer diagnosis has variable effects on patients depending on their age. Younger patients have an increased risk of depression, anxiety, and intrusive thoughts due to the possible disturbances of body image with hair loss, weight fluctuation, and paleness. In the general population, being physically active is linked to enhanced quality of life and emotional well-being. Physical activity during and after breast cancer treatment is safe [4,8-10]. According to studies, combining aerobic exercises with resistance training for six to 12 weeks significantly reduces anxiety, fatigue, and depressive symptoms in cancer survivors. Previous studies using a 36-item Short-Form Health Survey (SF-36) to evaluate the quality of life of breast cancer patients after exercise have shown significant improvements in general health and mental health components, such as social functioning. In multiple other studies, physical functioning and muscle strength consistently improved after exercise; however, there was no significant change in patients' fatigue levels [1,11-16].

Exercise adherence in breast cancer is defined as the ability to meet the recommended exercise requirements and guidelines. According to the Alberta Physical Activity and Breast Cancer Prevention (ALPHA) Trial, exercise adherence is associated with improved quality of life [17-19]. Despite these benefits, breast cancer survivors have an estimated exercise adherence rate of 54% to 81%, and research has shown that breast cancer survivors experience a decrease of 11% in their total physical activity post-diagnosis when compared to pre-diagnosis [14,18].

In addition to the everyday barriers the general population faces, survivors are often faced with discouragement toward exercise by their family members and friends due to the misconception that cancer patients need more time to rest and recover. According to past studies, women limited their exercise due to the fear of overdoing it and a lack of confidence in the safety of exercise after a cancer diagnosis. A study has shown that a simple recommendation to exercise by a treating physician significantly increased physical activity among breast cancer survivors [14]. Physicians addressing the common barriers and misconceptions can encourage breast cancer survivors to incorporate more exercise into their daily routine.

It is well established in the previously mentioned studies that customized exercise programs tailored to individual needs, preferences, and health statuses can enhance adherence. However, the compliance rate with the associated physical performance component change was not studied when the exercise is explicitly recommended by the treating physician in the survivorship period. The goal of this study is to investigate the effect of a structured exercise program on breast cancer survivorship and quality of life when exercise is endorsed by the treating physician.

This article was previously presented as a poster at the 2025 National Consortium of Breast Centers on March 25, 2025.

## Materials and methods

This is a prospective observational study that included breast cancer patients aged 18 and above who had completed their active treatment between six and 12 months prior and were enrolled in a structured exercise program specifically endorsed by their primary oncologic physician. Exclusion criteria included patients younger than 18 or patients who were undergoing active cancer treatment at the time of the study or who self-enrolled in a structured exercise program without the recommendation of their primary treating physician.

After completing active cancer treatment, patients' primary oncologic physician emphasized the importance of exercise in breast cancer survivorship. Patients consented and enrolled in a structured exercise program offered by a third-party not-for-profit organization with no direct affiliation to the clinical practice.

This structured exercise program was based on a virtual platform that incorporated evidence-based exercises and adhered to the American College of Sports Medicine (ACSM) exercise guidelines for individuals with cancer. Before the program started, patients filled out a health history questionnaire with clinical demographics and a quality-of-life survey.

The study's primary outcome was the effect of structured exercise on breast cancer survivors' quality of life. The quality-of-life survey used in this study was the SF-36. There is adequate evidence that the SF-36 survey has acceptable reliability and validity. Numerous studies have demonstrated the usability and reliability of the SF-36 across various populations. It assessed physical activity, bodily pain, general health, social functioning, emotional role, and mental health [3,6]. The secondary outcomes of interest included compliance rates with completion of the exercise program and long-term symptom improvements.

Patients had weekly virtual exercise classes with their certified cancer fitness instructor. These virtual classes were recorded, allowing participants to repeat the videos as much as desired. Attendance was recorded weekly. The number of times the videos were repeated and any additional exercises done during the 10 weeks were self-recorded. Patients were subjectively evaluated at week seven by their fitness instructors to assess many aspects targeted by the exercises, such as flexibility, strength, balance, and resistance. Patients completed the post-exercise SF-36 survey after completing 10 weeks. No additional lab work or office visits were needed in this study.

Participants' responses on the SF-36 survey at the end of the 10 weeks were scored in the range of zero to 100. High scores indicate better health. The eight scale scores were described with mean and standard deviation before and after the exercise program. The within-participant change was calculated as the participants' after score minus the participants' before scale score and summarized across all participants with mean and 95% confidence interval (CI) [11-13]. CIs that do not include zero were considered significant changes. The statistical analysis of the data was performed using the SAS v9.4v software (SAS Inc., Cary, NC, US).

## Results

A total of five female breast cancer patients, with an average age of 57 years, consented to and completed the exercise program. Two patients were Caucasian, one was African American, one was Asian, and one was of unknown ethnicity. The study's outcome was based on the pre-exercise SF-36 survey, the post-exercise SF-36 survey, and the cancer-certified fitness instructor's week seven evaluation.

**Figure 1:**
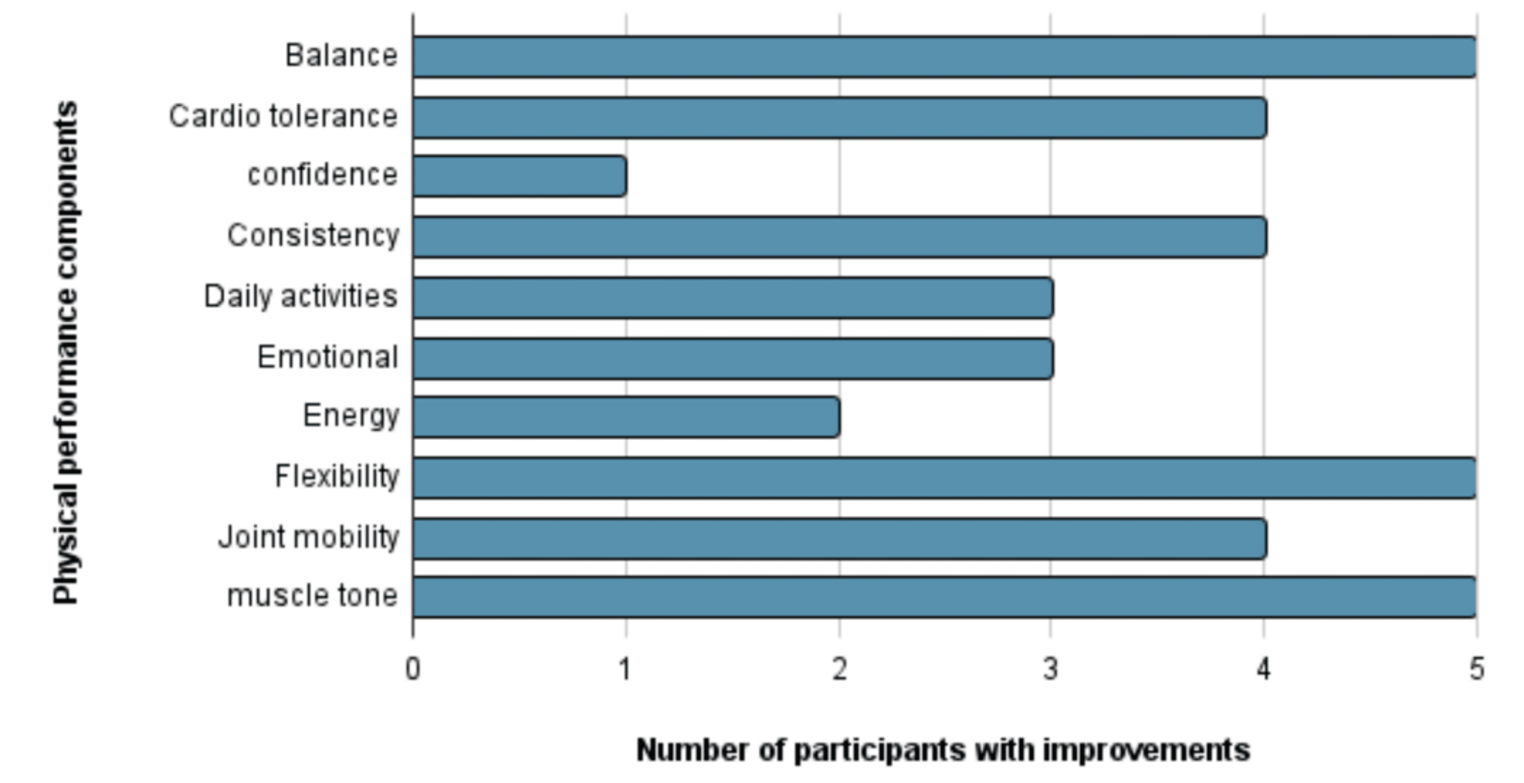
Number of participants with subjective improvements on week seven The number of participants with subjective improvements in balance, cardio tolerance, confidence, consistency, daily activity, emotional well-being, energy, flexibility, joint mobility, and muscle tone when evaluated by a fitness instructor on week seven.

Based on the instructor's week seven evaluation, there was an overall subjective improvement in performance, as summarized in Figure 1. All five participants had a noticeable improvement in flexibility, balance, and muscle tone.

Based on patients' responses on SF-36, there were significant improvements observed in their physical functioning (mean within-person change 13.0 points, 95% CI: 5.9 to 20.1, P < 0.05), energy/fatigue (mean change 16.0, CI: 2.5 to 29.5, P < 0.05), emotional well-being (mean change 9.6, CI: 1.3 to 17.9, P < 0.05), and social functioning (mean change 12.5, 1.5 to 23.5, P < 0.05). There was no significant mean change in participants' general health, pain level, limitation by physical health, and limitation by emotional problems, as summarized in Table 1.

**Table 1:** Mean change in quality-of-life scale components * indicates a two-sided Student t-test P-value < 0.05, and 95% CI (CL is the confidence limit) does not include zero.

Scale	N	Mean change (after – before)	Lower 95% CL for mean	Upper 95% CL for mean
Physical functioning*	5	13.0	5.9	20.1
Limits due to physical health	5	-18.8	-103.1	65.5
Limits due to emotional problems	5	20.0	-17.0	57.0
Energy/fatigue*	5	16.0	2.5	29.5
Emotional well-being*	5	9.0	1.3	17.9
Social functioning*	5	12.5	1.5	23.5
Pain	5	8.5	-10.2	27.2
General health	5	-9.0	-22.5	4.5

## Discussion

Our study highlighted significant improvements in emotional well-being, physical functioning, and social functioning, consistent with previous studies that have shown a link between physical activity and enhanced quality of life and emotional well-being [4,8-10]. In contrast to previous studies that showed no difference in fatigue levels, we demonstrated that both energy levels and fatigue improved with physician-endorsed structured exercise. In most of these earlier studies, the exercises were self-directed, not structured, nor endorsed by the treating physician, contrasting with our study [15]. We hypothesize that self-directed exercises can contribute to fatigue during the survivorship period, as patients are not instructed on the type and intensity of the exercises, which may explain the above differences.

Multiple studies have used the SF-36 to estimate the quality of life, covering eight main health concepts, such as physical functioning, bodily pain, role limitations due to physical health problems, role limitations due to personal or emotional problems, emotional well-being, social functioning, energy/fatigue, and general health perceptions [3,6,13]. Using this survey, we were able to compare the quality of life before and after exercise, demonstrating the significant improvements in quality-of-life components after exercise.

Additionally, we achieved a 100% compliance rate throughout the program. The participants described an increased sense of accountability and motivation. Previous studies have shown that breast cancer survivors have an estimated exercise adherence rate of 54% to 81% [14,18]. Based on our study, we hypothesize that patients may face fewer barriers and discouragement when the treating physician recommends structured exercise, which may lead to improved compliance. In the future, it would be interesting to investigate the effect of physician-endorsed structured exercise on compliance rate with exercise after completing the program and see whether the common misconceptions and barriers were addressed adequately during the exercise period.

Our pilot study only included female patients, which restricts the generalizability of the findings to other genders. This study has a limited but promising statistical power. The aforementioned inclusion and exclusion criteria limited our sample size and gender variability, as it was challenging to find patients who were enrolled in a structured program following a specific recommendation from their treating physician. Additionally, many structured exercise programs are not fully covered by medical insurance, unless in certain situations when prescribed by a healthcare provider. We predict that increased awareness among treating physicians will encourage enrollment and adherence, providing a larger sample size for future studies.

Our study demonstrated the feasibility of investigating the impact of a physician-endorsed structured exercise program on an expanded breast cancer population over a more extended period. Adding a control group in the future will help determine if any of the changes would have occurred regardless of the exercise program. The significant findings above are encouraging and represent the first step in developing a well-defined, physician-endorsed structured exercise program for breast cancer survivors.

## Conclusions

Participating in a structured exercise program in the survivorship period improves the quality of life of breast cancer patients. When regular exercise is specifically endorsed by a treating oncologic physician, there are high compliance rates, 100%, in this study. The study shows significant improvement in various components of quality of life, such as improved physical functioning, energy, fatigue, emotional well-being, and social functioning. It also demonstrated the feasibility of investigating the impact of a structured exercise program on breast cancer patients, with the aim of expanding it to a larger population size in the future for additional insights.
